# Mouse Adrenal Macrophages Are Associated with Pre- and Postsynaptic Neuronal Elements and Respond to Multiple Neuromodulators

**DOI:** 10.1523/ENEURO.0153-24.2025

**Published:** 2025-02-18

**Authors:** Matthew D. Whim

**Affiliations:** Department of Cell Biology & Anatomy, LSU Health Sciences Center, New Orleans, Louisiana 70112

**Keywords:** adrenal, calcium, chromaffin cells, GCaMP, neuroimmune, sympathetic

## Abstract

The adrenal medulla is packed with chromaffin cells, modified postganglionic sympathetic neurons that secrete the catecholamines, epinephrine and norepinephrine, during the fight-or-flight response. Sometimes overlooked is a population of immune cells that also resides within the gland but whose distribution and function are not clear. Here I examine the location of CD45+ hematopoietic cells in the mouse adrenal medulla and show the majority are F4/80+/Lyz2+ macrophages. These cells are present from early postnatal development and widely distributed. Anatomically they are associated with chromaffin cells, found aligned alongside synapsin-IR neuronal varicosities and juxtaposed to CD31-IR blood vessels. Using Lyz2cre-GCaMP6f mice to quantify calcium signaling in macrophages revealed these cells respond directly and indirectly to a wide variety of neuromodulators, including pre- and postganglionic transmitters and systemic hormones. Purinergic agonists, histamine, acetylcholine, and bradykinin rapidly and reversibly increased intracellular calcium. These results are consistent with a substantial resident population of innate immune cells in the adrenal medulla. Their close association with chromaffin cells and the preganglionic input suggests they may regulate sympatho-adrenal activity and thus the strength of the fight-or-flight response.

## Significance Statement

It is widely recognized that the nervous and immune systems can functionally communicate, but many aspects of the underlying cellular mechanisms remain to be clarified. The adrenal medulla contains neuroendocrine chromaffin cells and a diverse population of immune cells whose distribution and function is not well understood. Using immunohistochemistry, I show the medulla contains a large population of macrophages that are closely associated with the preganglionic input and with chromaffin cells. Monitoring calcium levels in macrophages within the mouse adrenal medulla shows these cells can rapidly respond to the application of a wide variety of neuromodulators. Their location and responsiveness suggests these cells could be involved in neuroimmune crosstalk during autonomic activation and the fight-or-flight response.

## Introduction

During the fight-or-flight response, sympathetic activation increases the activity of adrenal chromaffin cells and the release of the catecholamine hormones, epinephrine and norepinephrine, into the systemic circulation. For example, in response to hypoglycemia, autonomic activation and subsequent epinephrine release stimulates adipose tissue lipolysis and hepatic glycogenolysis and inhibits pancreatic insulin release (Cryer, 1993; Stanley et al., 2019).

Given the wide-ranging actions of the catecholamines, the activity of the chromaffin cells must be tightly regulated. The increase in chromaffin cell excitability during a stress response is controlled by the activity of sympathetic preganglionic neurons that innervate the adrenal medulla and which make excitatory, cholinergic synaptic connections onto chromaffin cells. Adrenal denervation or inhibition of cholinergic transmission prevents epinephrine release in response to multiple stressors (Lamarche et al., 1992; Zhou and Jones, 1993; Kvetnanský et al., 1996; McKinley et al., 2022). In addition, paracrine signaling pathways fine-tune chromaffin cell activity. Recent work has shown that an intra-adrenal feedback loop which involves the release of neuropeptide Y from chromaffin cells is required for sustained epinephrine release. Loss of this pathway likely contributes to HAAF, an activity-dependent impairment of epinephrine release observed in type I diabetes (Wang et al., 2016; Ma et al., 2018).

This suggests that additional signaling pathways that control epinephrine release, beyond the preganglionic input, remain to be identified. Possible candidates involve immune cells located in the adrenal gland. Early work identified macrophages in the rodent adrenal cortex and medulla (Hume et al., 1984; Sato, 1998; Schober et al., 1998; Engström et al., 2008) and macrophages are also present in the human adrenal (González-Hernández et al., 1994; Wang et al., 2023). While the function of these cells is not clear, in vivo injection of LPS results in a dramatic infiltration and reorganization of macrophages and dendritic cells throughout the rodent adrenal (Engström et al., 2008). A recent RNA-seq study demonstrated that the rodent adrenal contains a diverse population of immune cells in addition to macrophages, including T cells, B cells, dendritic cells, monocytes, and neutrophils (Dolfi et al., 2022). Systemic macrophage depletion reduces adrenal aldosterone synthesis (Dolfi et al., 2022) and in vitro, multiple cytokines including IL-1β and IFN-α can regulate adrenal catecholamine synthesis and release (Douglas and Bunn, 2009; Rosmaninho-Salgado et al., 2009). Finally, several studies have shown that activated macrophages, or macrophage-conditioned medium, can increase epinephrine secretion and modulate voltage-gated calcium channels in chromaffin cells (Jones et al., 1993; Roberts et al., 1996; Roberts and Jones, 1997; Lujan et al., 1998; Currie et al., 2000).

Although these studies are consistent with the role for immune cells in modifying adrenal output, whether these actions are mediated by cells that reside within the adrenal is unclear. This is particularly relevant in the context of the adrenal medulla, given the growing appreciation of crosstalk between the autonomic and immune systems (Pavlov et al., 2018; Klein Wolterink et al., 2022). Using a combination of immunohistochemistry and live cell imaging, I now show that the adrenal medulla contains a population of macrophages that are closely associated with chromaffin cells and the preganglionic input. Because these resident innate immune cells can respond to a variety of neuromodulators, this suggests they may be involved in modulating the sympatho-adrenal component of the fight-or-flight response.

## Materials and Methods

### Animals

C57BL/6J wild-type mice were bred from a laboratory colony. Lyz2cre mice (Clausen et al., 1999; JAX #004781), LSL-Salsa6f mice (Dong et al., 2017; JAX #031968), and NPYcre mice (Milstein et al., 2015; JAX #027851) were obtained from The Jackson Laboratory. Animals of both sexes were used. All experiments were approved by the Institutional Animal Care and Use Committee at Louisiana State University Health Sciences Center.

### Calcium imaging

To express the GCaMP6f calcium sensor in myeloid cells, Lyz2cre mice were crossed to LSL-Salsa6f mice. The Lyz2cre line is widely used for targeting macrophages but is also expressed in other immune cells including granulocytes (Hume, 2011). Adrenal glands were isolated from male and female Lyz2cre-GCaMP6f-tdTomato mice that were killed by decapitation [postnatal days (P) 22–P56]. Adrenal sections (100 µm) were prepared largely as previously described (Wang et al., 2016). In brief, the glands were embedded in low melting point agarose, and the tissue blocks were trimmed and then mounted on a Leica VT100 Vibratome. Sections were cut in ice-cold ACSF (artificial cerebral spinal fluid; in mM: 125 NaCl, 3 KCl, 2 CaCl_2_, 1 MgCl_2_, 25 NaHCO_3_, 1.25 NaH_2_PO_4_, 5.5 glucose, pH 7.4) that was bubbled with 95% O_2_/5% CO_2_. The sections were subsequently transferred to the same solution maintained at room temperature (22–24°C). To monitor calcium activity, a single adrenal slice was transferred to an imaging chamber on the stage of a Nikon TE2000U inverted microscope. The chamber (volume ∼600 µl) was continually superfused at ∼3 ml/min with extracellular solution (in mM: 135 NaCl, 3 KCl, 2 CaCl_2_, 1 MgCl_2_, 10 HEPES, 5.5 glucose, pH 7.4) at room temperature. In some experiments (Fig. 7*D*), the extracellular solution contained 0 mM CaCl_2_ and 2 mM EGTA. Slices were viewed with a 20× objective and illuminated using a DG-4 xenon light source (Sutter Instrument). A region of the medulla containing macrophages was identified by RFP (red fluorescent protein) expression in Lyz2cre-GCaMP6f-tdTomato mice (excitation 530–550 nm/emission 590–650 nm) and positioned in the center of the field of view. The GCaMP6f signal (excitation 460–500/emission 510–560 nm) was collected (500 ms exposure time) every 5 s using NIS-Elements (Nikon Imaging Software). To quantify changes in [Ca^2+^]_i_, ROIs were drawn to encircle 10–15 individual cells identified in the RFP channel, and the mean fluorescence change was then quantified in the GFP channel. This approach avoided inadvertently biasing the data collection to cells that showed the largest change in calcium levels. Fiji/ImageJ (Schindelin et al., 2012), Clampfit10 (Molecular Devices), Origin 2019b (OriginLab), and Excel were used for data analysis. In each figure the number of biological replicates (*n* = number of mice) is noted. Changes in GCaMP6f signal in response to agonist application were performed on raw data and calculated as *F*t − *F*0 (fluorescence at time *t* − fluorescence immediately before agonist application). For example recordings (Figs. 6, 7), slow changes in baseline signal over the course of an experiment were removed using Clampfit.

### Additional tissue and cell type isolation from Lyz2cre-GCaMP6f-tdTomato mice

(1) Adrenal cells (both cortex and medulla) were dissociated using enzymatic digestion as previously described for chromaffin cells (Whim, 2006). Dissociated cells were plated on poly-d-lysine coated coverslips in DMEM/10% FCS. After allowing 1 h for attachment, the cells were subsequently used within 6 h of isolation. (2) Peritoneal macrophages were collected from euthanized mice by peritoneal lavage with 3 ml of DMEM/10% FCS. After centrifugation (1,000 rpm for 3.5 min), the pellet was resuspended in DMEM/10% FCS, and cells were plated on uncoated coverslips and used within 6 h. Staining indicated most cells in these cultures were F4/80-IR (83 ± 3%, mean ± SEM, 76 cells from *n* = 3 mice). (3) Lung tissue slices were prepared using the same approach as described for the adrenal gland. Small pieces of pulmonary tissue were dissected from the base of the right lung of euthanized mice and embedded in low melting point agarose. After isolation, tissue sections (100 µm) were maintained at room temperature in ACSF bubbled with 95% O_2_/5% CO_2_. Because both pulmonary macrophages and alveolar type 2 (epithelial) cells express lysozyme 2 (and thus GCaMP6f), macrophages were identified as those cells which showed an increase in [Ca^2+^]_i_, in response to 100 µM nicotinic acid, a HCAR2 (hydroxycarboxylic acid receptor 2) agonist. The LungMAP RNA-seq dataset (lungmap.net) indicates HCAR2 is strongly expressed by alveolar macrophages but not alveolar type 2 cells.

### Immunohistochemistry

Adrenal glands were isolated from male and female C57BL/6J wild-type and Lyz2cre-GCaMP6f-tdTomato mice that were killed by decapitation. Glands were fixed in 4% paraformaldehyde for 1 h at 4°C, then washed with PBS, and transferred to PBS/30% sucrose for 24 h at 4°C. Glands were snap frozen in 2-methylbutane on dry ice, embedded in OCT (Optimal Cutting Temperature compound), and 30 μm cryosections were prepared and stored in cryoprotectant (Watson et al., 1986) at −20°C until used. For staining, sections were removed from cryoprotectant, washed with PBS (containing 0.05% Triton X-100),and then incubated sequentially in 3% H_2_O_2_ and 2 mg/ml sodium borohydride (each for 20 min) at room temperature. Sections were incubated shaking overnight at 4°C or at room temperature in primary antibody (diluted in PBS/1% IgG-free BSA/0.3% Triton). Next day, the sections were washed with PBS. For TSA (Tyramide Signal Amplification) staining, sections were incubated in HRP-coupled secondary antibodies (1:250 diluted in PBS/1% IgG-free BSA/0.05% Triton) for 30 min at room temperature. After washing, the sections were incubated in 1:50 TSA-FITC or TSA-TMR reagent following the manufacturer's instructions (PerkinElmer), then washed, and mounted in Vectashield (VectorLabs). For regular indirect immunofluorescence, after overnight incubation in primary antibody, the sections were washed, incubated for 90 min at room temperature in 1:100 fluorescently labeled secondary antibody, and then washed in PBS and mounted in Vectashield. Before mounting, all sections were incubated in DAPI for 15 min at room temperature (1 µg/ml in PBS/0.05% Triton) to stain cell nuclei. Primary antibodies were as follows: rat anti-CD45 (1:10,000, BioLegend BL103101, RRID AB_312966); rat anti-F4/80 (1:5,000, BioLegend BL123101, RRID AB_893504); rat anti-CD68 (1:20,000, BioLegend BL137001, RRID AB_ 2044003); rat anti-CD301 (1:5,000, Abd Serotec MCA2392, RRID AB_872014); rat anti-CD115 (1:5,000, BioLegend BL135501, RRID AB_1937292); rat anti-MHC II (1:10,000, BioLegend BL107601, RRID AB_313316); rat anti-Iba1 (1:2,000, Cell Signaling Technology CST17198, RRID AB_2820254); rabbit anti-RFP (1:1,000, Rockland 600-401-379, RRID AB_2209751); chicken anti-RFP (1:20,000, Rockland 600-901-379, RRID AB_10704808); rabbit anti-tyrosine hydroxylase (1:2,000, Cell Signaling Technology CST2792, RRID AB_2303165); guinea pig anti-PNMT (1:200, Acris EDU7001, RRID AB_1006533); rabbit anti-NPY (1:10,000, Peninsula T4070, RRID AB_518504); rabbit anti-GFAP (1:200, Dako Z0334, RRID AB_10013382); rabbit anti-MAP2 (1:500, Millipore AB5622, RRID AB_91939); rat anti-CD31 (1:200,000, BioLegend BL102501, RRID AB_312908); rabbit anti-acetylated tubulin (1:2,000, Cell Signaling Technology CST5335, RRID AB_10544694); rabbit anti-synapsin (1:500, Millipore AB1543, RRID AB_2200400). Secondary antibodies (all from Jackson ImmunoResearch) were the following: donkey anti-rat HRP (1:250); donkey anti-chicken HRP (1:1,000); donkey anti-rabbit Cy3 (1:100); donkey anti-rabbit Alexa 488 (1:100), and donkey anti-guinea pig FITC (1:100). Images were obtained with a Nikon TE2000U inverted microscope with 10× and 40× objectives and a DG-4 xenon light source. When using the 40× objective, *z*-stacks (11 images collected in the *z*-plane with a 1.2 µm step size) were deconvolved using NIS-Elements (Nikon) software (using blind 3D deconvolution with a theoretical point spread function and no background subtraction). This was adopted because even in 30 µm cryosections, some out-of-focus fluorescence was present (medulla macrophages are thin cells with wandering processes that do not lie in the horizontal plane). However, all quantitative measurements were made on data obtained with the 10× objective and these were not deconvolved.

To quantify the degree of colocalization (Fig. 6*A*), a threshold criterion was used to identify stained cells. This was met by cells whose mean fluorescence intensity exceeded the “background + 2×SD” as previously described (Glynn and McAllister, 2006). In brief, background was first quantified individually for each stained section by placing ROIs around 10 clearly stained cells in each fluorescence channel. Each ROI was then displaced adjacent to the stained cell (avoiding cell-free areas such as medulla sinusoids) and the mean intensity + (2×SD) was calculated. Any cell in the medulla whose level of fluorescence exceeded this value was then scored as positive. The advantage of this method is that background fluorescence is measured on a slide-by-slide basis. However, other approaches (e.g., staining wild-type mice with RFP primary and secondary antibodies) confirmed that the background was low (Extended Data Fig. 6-1*A–D*). Figures were assembled using Fiji and Inkscape.

### Statistical tests

Comparisons between groups that contained normally distributed data (assessed using the Shapiro–Wilk test) were made using analysis of variance (Tukey post hoc). The Kruskal–Wallis ANOVA (Dunn's post hoc) was used for non-normal data. Origin 2019b and Excel were used for analysis. All values are mean ± standard error of the mean (SEM) and *p* < 0.05 was considered significant. Table 1 lists the tests used in this study.

**Table 1.** Statistical tests used for data analysis

Data structure	Type of test	Power
Figure 5*G*	Non-normal; Kruskal–Wallis ANOVA	NS
Figure 6*B*	Normal distrib; one-way ANOVA	0.05
Figure 6*C*	Normal distrib; one-way ANOVA	0.01–0.001
Figure 7*C*, pyrilamine	Normal distrib; one-way ANOVA	0.5–0.01
Figure 7*C*, cimetidine	Non-normal; Kruskal–Wallis ANOVA	NS
Figure 7*D*, rapid	Normal distrib; one-way ANOVA	0.5
Figure 7*D*, slow	Non-normal; Kruskal–Wallis ANOVA	NS

## Results

### Macrophages are widely distributed throughout the adrenal medulla

**Figure 1.**
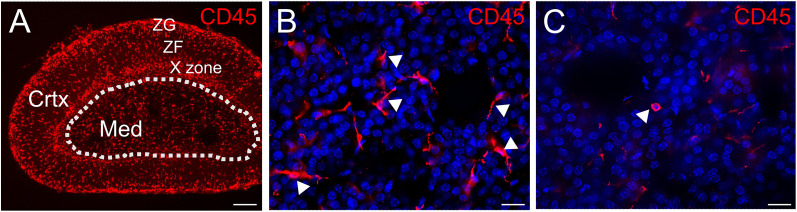
Immune cells are distributed throughout the mouse adrenal gland. ***A***, CD45-immunoreactive myeloid cells are present in the cortex (Crtx) and medulla (Med) of the adrenal gland. ***B***, In the medulla, the majority of CD45-IR cells are elongated with fine processes. ***C***, An example of a rare spherical CD45-IR cell in the medulla. ZG, zona glomerulosa; ZF, zona fasciculata. Scale bar: 200 µm (***A***); 20 µm (***B***, ***C***).

**Figure 2.**
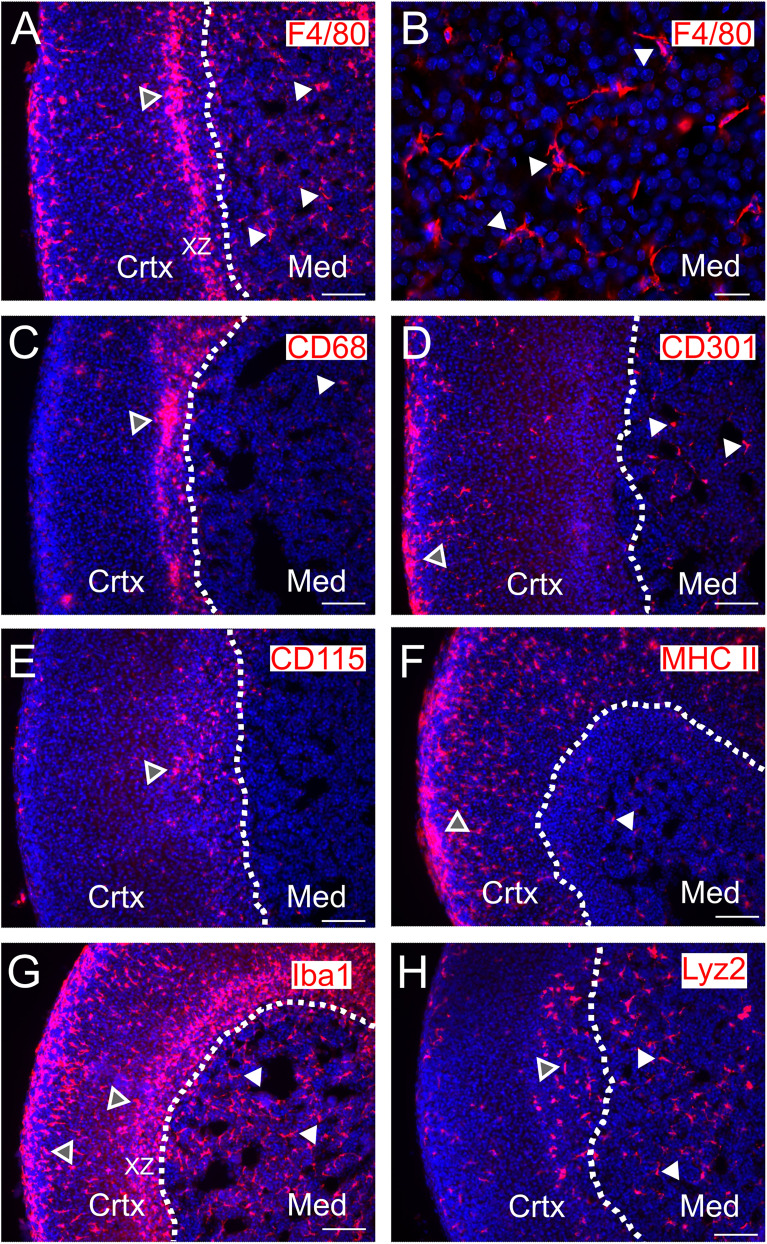
Cells in the adrenal medulla are labeled with a variety of macrophage markers. ***A***, F4/80-IR cells are present in the adrenal cortex (Crtx) and medulla (Med). F4/80-IR labels a band of cells in the cortical X-zone (xz; open arrowhead) and cells throughout the medulla (closed arrow heads). ***B***, Higher magnification of F4/80-IR cells in the medulla indicates the cells are elongate with fine processes. ***C***, CD68-IR cells are prominent in the X-zone and also present in the medulla. ***D***, CD301-IR cells are located in the capsule/zona glomerulosa and medulla. ***E***, CD115-IR cells are located in the cortex and sparse in the medulla. ***F***, MHC II-IR cells are primarily located in the outer cortex. ***G***, Macrophages that are Iba1-IR are present throughout the adrenal including the medulla. ***H***, Lysozyme2-expressing cells (Lyz2) labeled with tdTomato (Lyz2cre-GCaMP6f mouse) are present in an inner cortical region and throughout the adrenal medulla. Scale bar: 100 µm (***A***, ***C–H***); 20 µm (***B***). See also Extended Data Figure 2-1 for more details.

To visualize immune cells in the adrenal, cryosections were stained for expression of CD45, which labels all hematopoietic cells. CD45-IR cells were present throughout all adrenal zones (Fig. 1*A*). Within the medulla, the majority of CD45-IR cells had numerous fine processes (Fig. 1*B*) although occasional spherical cells were also found (Fig. 1*C*). Consistent with a significant adrenal population of macrophages, many F4/80-IR cells were present, including within the medulla (Fig. 2*A*,*B*). The X-zone, a cortical zone that degenerates postnatally in mice (Hershkovitz et al., 2007), was strongly immunoreactive (Fig. 2*A*).

10.1523/ENEURO.0153-24.2025.f2-1 Figure 2-1 **Macrophages in the adrenal medulla.** A-C. Adrenal cryosections from a wild type mouse co-stained for Iba1 and F4/80. D. Distribution of fluorescence intensities for Iba1-ir cells fit with a single Gaussian distribution. E. Distribution of F4/80-ir fluorescent signal. F. Group data showing that Iba1- and F4/80-ir cells in the medulla are co-localized (mean ± SEM, n = 6 mice). Scale bar 50  µm. Download Figure 2-1, TIF file.

Macrophages are molecularly and functionally plastic cells. Historically they have been separated into M1 and M2 populations (classically activated and proinflammatory vs alternatively activated and anti-inflammatory, respectively). It is now recognized that macrophage state exists on a continuum with their physiology being determined by developmental origin, environmental context, and tissue location (Katkar and Ghosh, 2023). I next selected a variety of commonly used macrophage markers to examine the distribution of these cells with particular attention to those located in the medulla. Staining for CD68 (a lysosomal associated membrane protein) and CD301 (Clec10A, C-type lectin domain family 10A) labeled cells within the cortex but only a modest number of cells in the medulla (Fig. 2*C*,*D*). Immunoreactivity for the CSF1 receptor (CD115) which is differentially expressed in tissue macrophages labeled very few cells in the medulla (Fig. 2*E*). MHC II (major histocompatibility II), a marker of antigen-presenting cells, showed labeling throughout the cortex with lower staining in the medulla (Fig. 2*F*). In contrast, Iba1-IR (ionized calcium binding adaptor 1) which is highly expressed by microglia showed intense staining in all adrenal zones (Fig. 2*G*). Staining adrenal sections for both F4/80 and Iba1 showed that the majority of cells were colabeled (Extended Data Fig. 2-1) indicating that they identified the same population of macrophages. Finally, using a Lyz2cre transgenic line which is widely used to identify macrophages (and monocytes) revealed that many Lyz2-expressing cells were present in the medulla (Fig. 2*H*). Thus, there is an extensive population of tissue-resident macrophages in the rodent adrenal medulla, as previously reported (Hume et al., 1984; Sato, 1998; Schober et al., 1998; Engström et al., 2008; Dolfi et al., 2022). Two recent RNA-seq studies have profiled mouse adrenal cells and identified three (Bedoya-Reina et al., 2021) or four (Dolfi et al., 2022) populations of adrenal macrophages. Comparing the staining profile in Figure 2 with transcript expression in these studies indicated it was not possible to categorically assign medulla macrophages. For example, Iba1 was highly expressed in all three macrophage populations in Bedoya-Reina et al. (2021); restricted to cluster #2 in Dolfi et al. (2022) and immunohistochemically labeled cortical and medulla macrophages (Fig. 2*G*).

### Macrophages are associated with pre- and postganglionic cells in the adrenal medulla

**Figure 3.**
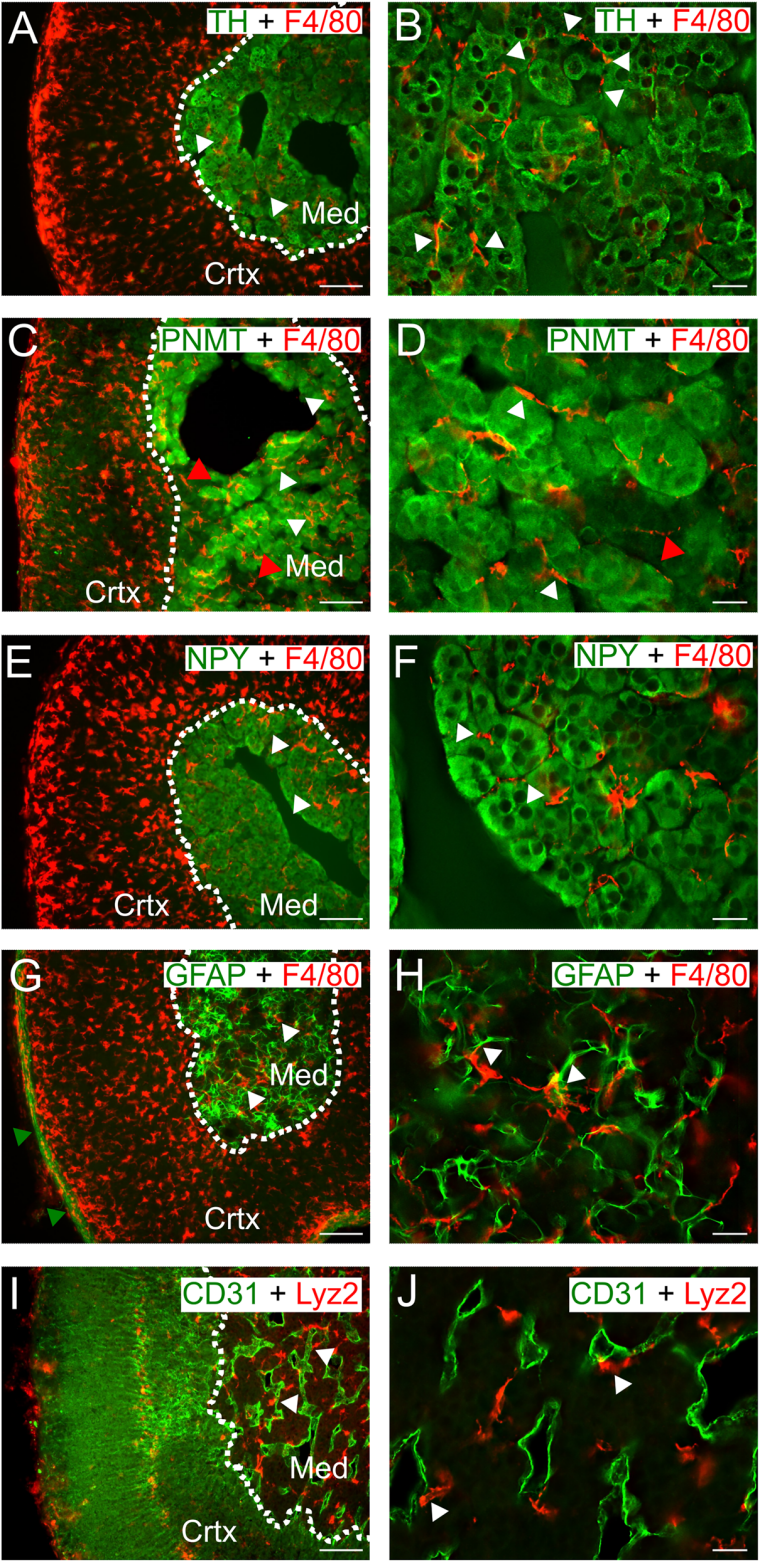
Macrophages in the adrenal medulla are closely associated with multiple cell types. ***A***, ***B***, F4/80-IR cells in the medulla are intermingled with tyrosine hydroxylase-IR chromaffin cells. ***C***, ***D***, F4/80-IR macrophages are juxtaposed to PNMT-IR (white arrowheads) and PNMT-negative chromaffin cells (red arrowheads). ***E***, ***F***, F4/80-IR cells are located next to chromaffin cells that are NPY-IR. ***G***, ***H***, F4/80-IR cells (white arrowheads) are close to GFAP-IR satellite glial cells in the medulla. Note also a band of GFAP-IR cells is located in the adrenal capsule (green arrowheads). ***I***, ***J***, Some Lyz2-expressing cells (white arrowheads) are close to CD31-IR endothelial cells in the medulla. Scale bar: 100 µm (***A***, ***C***, ***E***, ***G***, ***I***); 20 µm (***B***, ***D***, ***F***, ***H***, ***J***). See also Extended Data Figure 3-1 for more details.

To determine whether the medulla macrophages are present in discrete anatomical niches, the colocalization of F4/80-IR and markers of other medulla cell types was examined. The rationale for selecting the F4/80 antibody was that the F4/80 (Adgre1) antigen is the most widely used marker of tissue-resident macrophages and the Adgre1 transcript is reportedly present in all adrenal macrophages (Dolfi et al., 2022). F4/80-IR cells were closely associated with chromaffin cells that were visualized with TH-IR (Fig. 3*A*,*B*). Macrophages were juxtaposed to both PNMT positive and negative cells indicating that the immune cells were associated with both epinephrine- and norepinephrine-secreting chromaffin cells (Fig. 3*C*,*D*). NPY is a peptide cotransmitter that is expressed by all mouse chromaffin cells (Wang et al., 2013), and F4/80-IR macrophages were apposed to NPY-IR cells as expected (Fig. 3*E*,*F*). GFAP-IR, a marker of satellite glial cells (Avraham et al., 2020; Hanani and Spray, 2020), was restricted to the medulla and the adrenal capsule. Costaining with F4/80 and GFAP showed macrophages and glial cells were often juxtaposed (Fig. 3*G*,*H*). Finally, labeling with CD31 to visualize the endothelial cells that line blood vessels revealed that some Lyz2-expressing macrophages also extended processes that appeared to contact blood vessels within the medulla (Fig. 3*I*,*J*).

10.1523/ENEURO.0153-24.2025.f3-1 Figure 3-1 **Macrophages in the adrenal medulla are close to multiple adrenal cell types.** A. F4/80-ir macrophages intermingle with TH-ir chromaffin cells (left panel). In the orthogonal view (middle panel), areas of close juxtaposition (arrow heads) are seen. Line scans of fluorescence intensity normalized to peak intensity in each channel (right panel). B. F4/80-ir macrophages and GFAP-ir satellite glial cells. C. Some RFP-ir macrophages in a Lys2-cre-GCaMP6f-tdTomato mouse are close to CD31-ir endothelial cells. D. Some F4/80-ir macrophages are aligned along acTub-ir neuronal processes. In A-D, orthogonal views are taken from the white boxed areas shown in the corresponding left panels. Line scans were measured along the yellow vertical line shown in each Y-Z projection. Scale bar 20  µm (left panels); 5  µm (z axis, middle panels). Download Figure 3-1, TIF file.

**Figure 4.**
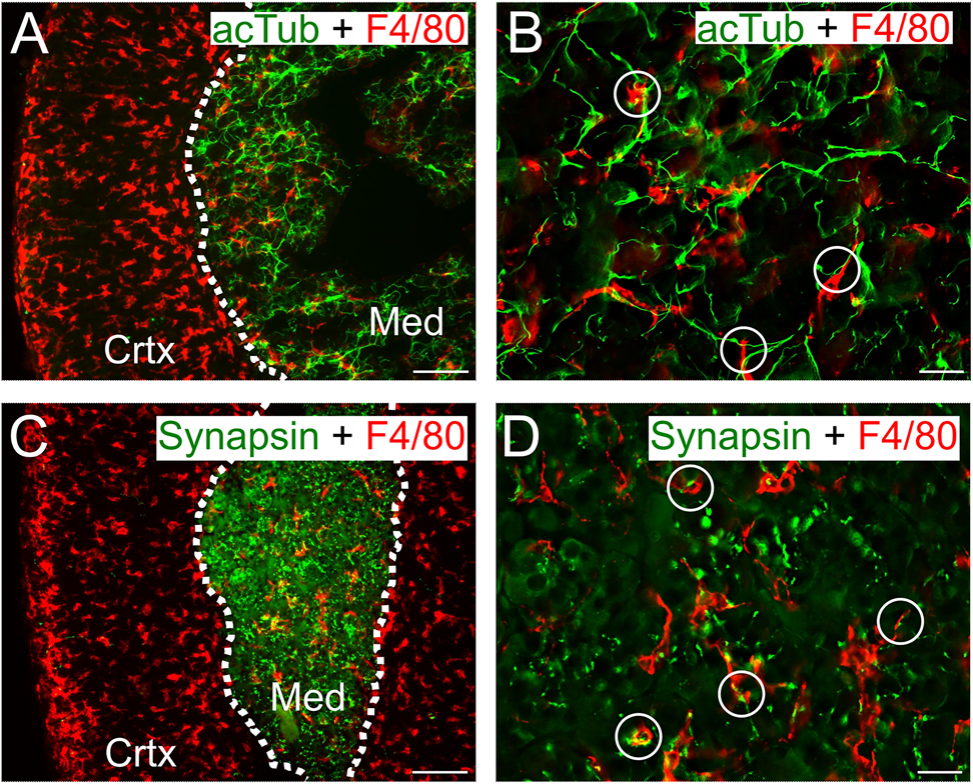
Nerve-associated macrophages in the adrenal medulla. ***A***, ***B***, Staining for an axonal marker (acetylated tubulin, acTub) reveals dense labeling throughout the medulla. Colocalization with F4/80-IR shows that macrophages are associated with acTub-IR axonal processes and often aligned (white circles). ***C***, ***D***, Synapsin-IR synaptic varicosities are densely distributed throughout the adrenal medulla. Higher magnification reveals potential sites of interaction with F4/80-IR macrophages in the medulla (white circles). Scale bar: 100 µm (***A***, ***C***); 20 µm (***B***, ***D***).

Recent studies have identified a population of macrophages that can functionally interact with peripheral autonomic nerves (Pirzgalska et al., 2017; Wolf et al., 2017; Ural et al., 2020). To determine whether macrophages are associated with the preganglionic input to the adrenal, cryosections were stained for acetylated tubulin which is enriched in axons (Cambray-Deakin and Burgoyne, 1987). F4/80-IR cells were in close contact with acTub-IR processes and often aligned alongside acTub-IR structures (Fig. 4*A*,*B*). To confirm the association of macrophages and neuronal processes, I next stained for synapsin, a synaptic vesicle protein. Many F4/80-IR cells were juxtaposed to synapsin-IR puncta (Fig. 4*C*,*D*). The adrenal also receives vagal afferent and efferent input (Coupland et al., 1989), sensory innervation from dorsal root ganglia (Mohamed et al., 1988; Zhou et al., 1991), and limited postganglionic input (Kesse et al., 1988). However, the acTub- and synapsin-IR structures likely reflect the dense innervation of the medulla by cholinergic sympathetic preganglionic neurons (Kesse et al., 1988; Gautron et al., 2013). Examination of *z*-stacks confirmed the close association between macrophages and chromaffin cells, glial cells, endothelial cells, and axons (Extended Data Fig. 3-1).

**Figure 5.**
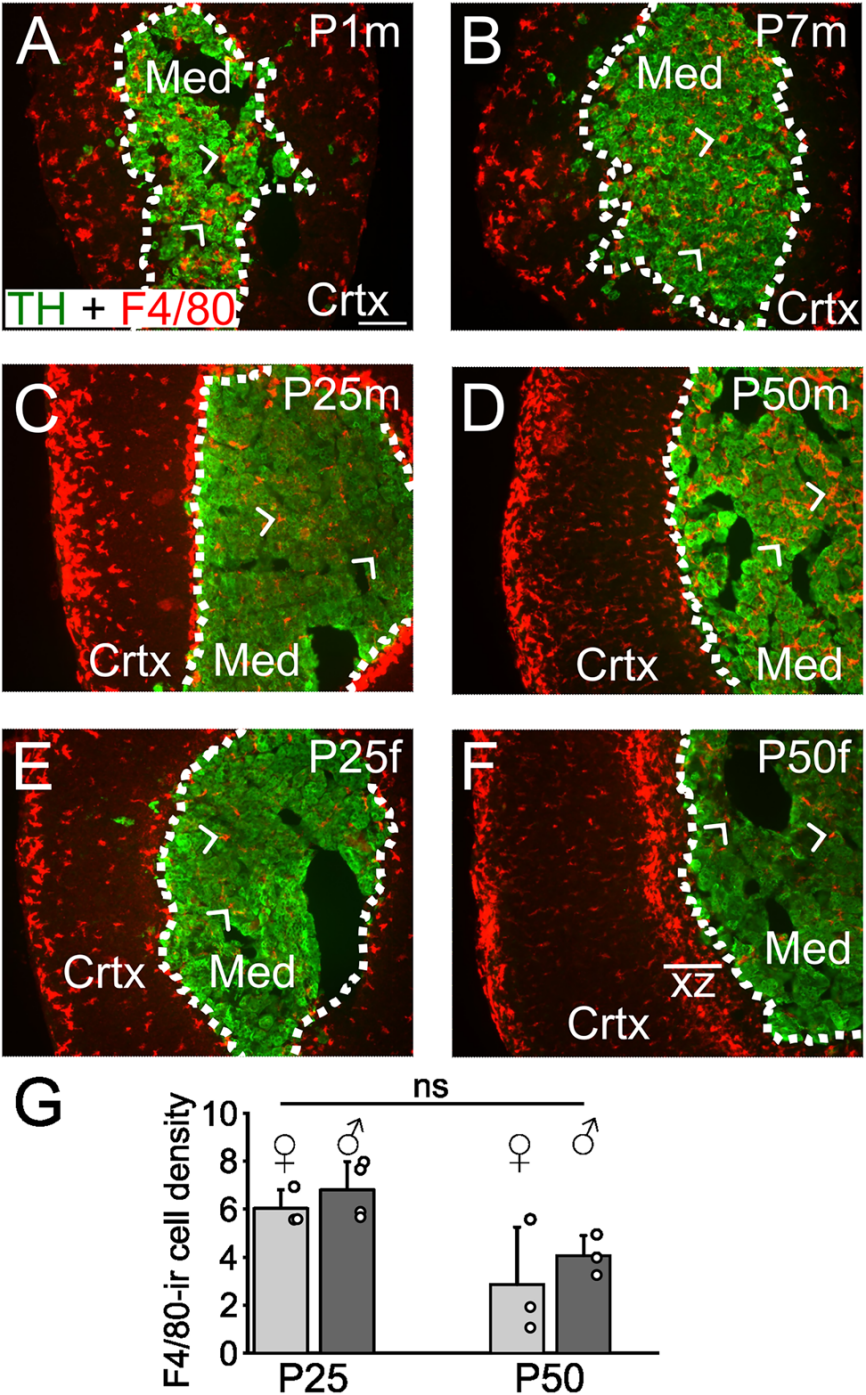
Macrophages are present in the adrenal medulla throughout postnatal development. ***A−D***. Examples of adrenal cryosections showing that F4/80-IR macrophages (arrow heads) are present throughout the adrenal cortex and medulla (TH-IR zone) in male mice at postnatal days 1 to 50. ***E***, ***F***, F4/80-IR macrophages in the cortex and medulla of female mice. ***G***, Group data showing the relative density of F4/80-IR cells in the adrenal medulla at P25 and P50 (mean #/0.01 mm^2 ^± SD, *n* = 3–4 mice). Scale bar, 100 µm.

Finally, to determine whether adrenal macrophages are present throughout postnatal development, I examined the presence of F4/80-IR cells in neonatal (P1) mice and at P7, P25, and P50. Macrophages were evident in the medulla at all time points (Fig. 5*A−D*) and in both sexes (Fig. 5*C−F*). Although there was a tendency for fewer F4/80-IR cells at P50, this was not statistically significant (Fig. 5*G*). There was also no difference in the number of Iba1-IR cells in the medulla of female and male at P50 (6.05 ± 0.53 vs 6.7 ± 0.84, mean #/0.01 mm^2 ^± SD, *n* = 3 mice, *p* = 0.31).

In sum, these findings indicate the presence of a large population of tissue-resident macrophages in the mouse adrenal medulla. Although these cells do not appear to be restricted to particular regions within the medulla, they are anatomically close to both pre- and postsynaptic neuronal components (preganglionic neurons and chromaffin cells, respectively). One implication of this physical arrangement is the existence of adrenal neuroimmune crosstalk.

### Multiple GPCRs are coupled to [Ca^2+^]_i_ release in macrophages in the adrenal medulla

**Figure 6.**
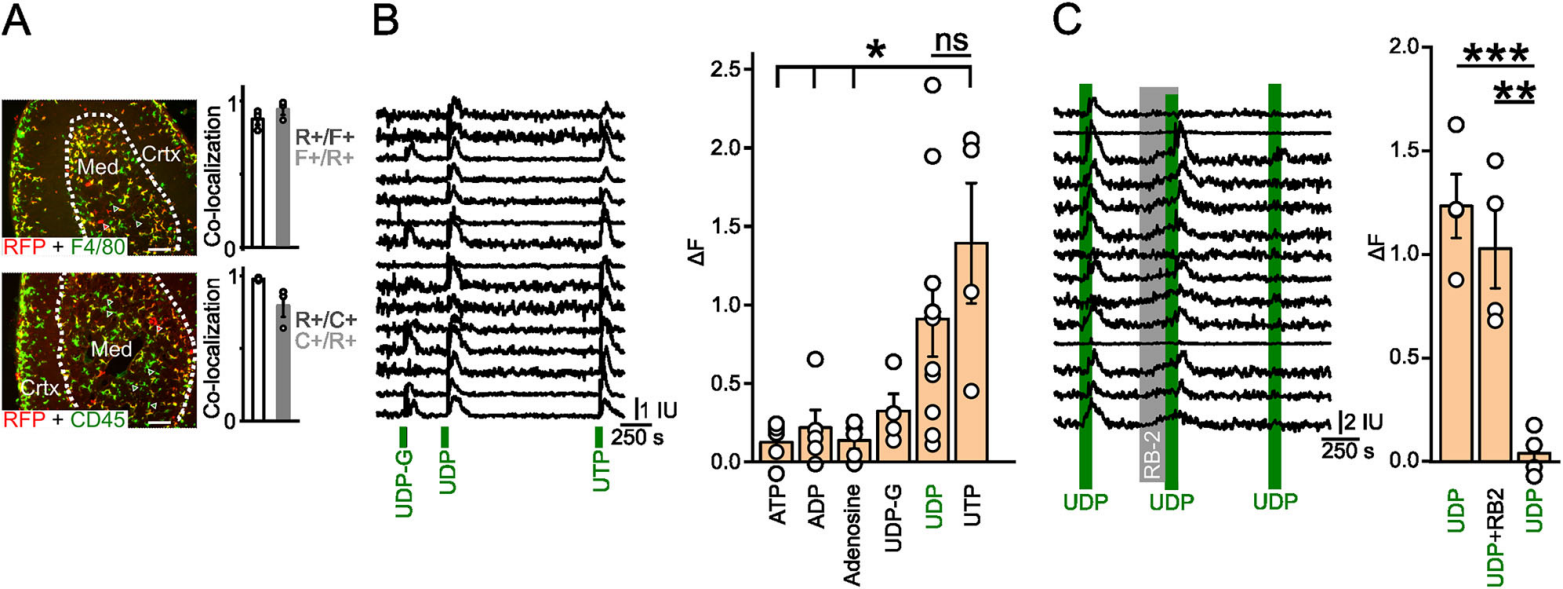
Adrenal macrophages express P2Y purinergic receptors. ***A***, Adrenal cryosections from a Lyz2cre-GCaMP6f-tdTomato mouse, costained for RFP and F4/80 (top panel) and RFP and CD45 (bottom panel). Quantification of immunoreactivity within the medulla (*n* = 3 mice, 67–171 cells per mouse) shows that RFP-IR colocalizes with F4/80- and CD45-IR (R+/F+ and R+/C+, respectively). Most F4/80- and CD45-IR also colocalizes with RFP-IR (F+/R+ and C+/R+, respectively). ***B***, Example of GCaMP6f fluorescent signal in medulla macrophages in a single adrenal slice from a Lyz2cre-GCaMP6f-tdTomato mouse (left panel, IU, intensity units). Application of 100 µM UDP-glucose, UDP, and UTP is indicated by the green bars. Group data (right panel) showing the response to a panel of P2Y agonists (each applied at 100 µM, mean ± SEM, *n* = 4–10 mice, each open symbol is the average of 15 cells per mouse). ***C***, Calcium increase in response to 100 µM UDP is blocked following application of 100 µM reactive blue-2 (left panel). Group data (right panel; mean ± SEM, *n* = 4 mice, 15 cells per mouse). *p < 0.05, **p < 0.01, ***p < 0.001. Scale bar, 100 µm. See also Extended Data Figures 6-1, 6-2 for more details.

An emerging feature of macrophage biology is the realization that tissue-resident macrophages are adapted to their local environment. For example, peripheral nerve-associated macrophages (NAMs) have a transcriptional profile that distinguishes them from macrophages in the CNS or peritoneal cavity (Wang et al., 2020; Gainullina et al., 2023). This suggests that medulla macrophages might respond to modulators relevant to autonomic function. With this in mind, I monitored the levels of intracellular calcium in medulla macrophages in adrenal slices and quantified the response to agonist application. Adrenal sections were prepared from Lyz2cre-GCaMP6f-tdTomato mice. Cells expressing the GCaMP6f calcium sensor (identified by the coexpression of tdTomato) were present throughout the adrenal. To determine which medulla cell types were labeled, adrenal sections were costained for RFP and immune cell markers. This showed that 98% of RFP-IR cells in the medulla were CD45-IR, indicating that RFP (and thus GCaMP6f) expression was restricted to immune cells. Conversely, 80% of CD45-IR cells were RFP positive (Fig. 6*A*). Thus, there are some immune cells in the medulla that do not express Lyz2-cre, possibly T cells or B cells (Dolfi et al., 2022). Next, to examine what fraction of the RFP cells were macrophages, sections were costained for RFP and F4/80 (Fig. 6*A*). Quantification of the staining indicated that 88% of the RFP-IR cells in the medulla were F4/80-IR. Conversely, 98% of medulla F4/80-IR cells were also RFP positive. Thus, all medulla macrophages/monocytes express RFP/GCaMP6f (Fig. 6*A*), but there is a small population of Lyz2-cre positive cells (∼12%) that express RFP/GCaMP6f but are F4/80 negative. The identity of these Lyz2-cre expressing cells is not known but could include granulocytes, dendritic cells, or innate lymphoid cells (Shi et al., 2018; Dolfi et al., 2022).

10.1523/ENEURO.0153-24.2025.f6-1 Figure 6-1 **Controls for specific labelling of immune cells in the mouse adrenal gland.** A. RFP-immunoreactive cells in the adrenal gland of a Lys2-cre-GCaMP6f-tdTomato mouse. B. Higher power image showing RFP-ir cells (arrow heads) in the adrenal medulla. C. RFP-ir in the adrenal gland of a wild type mouse. D. Higher power view of the adrenal medulla showing that background levels of fluorescence are low. Thus, fluorescence signal in A,B does not arise from non-specific secondary antibody staining. E. RFP-ir in the adrenal gland of a NPYcre-GCaMP6f-tdTomato mouse. F. RFP-ir cells in the adrenal medulla with the characteristic morphology of chromaffin cells (arrow heads). No cells with a macrophage-like morphology (compare with B) are seen. Scale bar 200  µm (A,C,E); 20  µm (B,D,F). Download Figure 6-1, TIF file.

10.1523/ENEURO.0153-24.2025.f6-2 Figure 6-2 **No neuronal expression of GCaMP6f is seen in the adrenal medulla of Lyz2cre-GCaMP6f-tdTomato mice.** A. RFP-ir in the adrenal medulla of a Lyz2cre-GCaMP6f-tdTomato mouse. B. MAP2-ir (a neuronal marker) in the same cryosection is present in cells throughout the adrenal medulla (presumably chromaffin cells and intra-adrenal ganglion neurons) and in cell processes in the capsule and cortex (arrow heads). C. Merged image. D-F. In the adrenal medulla, higher power images show that RFP-ir cells (white arrowhead) do not co-localize with MAP2-ir cells (filled arrowhead) or MAP2-ir processes (open arrowhead). Combined with other data (Fig 2–4, 6) this indicates that RFP (and thus GCaMP6f) is expressed in macrophages and not neuronal cells. Scale bar 200  µm (A,B,C); 20  µm (D,E,F). Download Figure 6-2, TIF file.

Lyz2-cre expression has also been reported in some neuronal cells (Orthgiess et al., 2016). To determine if this occurred in the adrenal, sections were costained for the neuronal marker, MAP2 and RFP. As shown in Extended Data Figure 6-2, in addition to the neuroendocrine chromaffin cells which are MAP2-IR, the adrenal contained MAP2-IR processes in the capsule, cortex, and medulla. The processes in the medulla are likely to arise from intra-adrenal ganglion neurons or sensory afferents (Mohamed et al., 1988; Dagerlind et al., 1995). However, the MAP2-IR did not colocalize with RFP. Finally, to directly express GCaMP6f in chromaffin cells, I generated an NPYcre-GCaMP6f-tdTomato mouse line (NPY is expressed by all mouse chromaffin cells; Wang et al., 2013). As shown in Extended Data Figure 6-1*E,F*, RFP was present throughout the medulla in cells with the distinctive morphology of chromaffin cells. Thus, in the adrenal medulla of Lyz2cre-GCaMP6f-tdTomato mice, RFP (and GCaMP6f) expression is effectively restricted to hematopoietic (CD45-IR) cells and is not present in neurons or neuroendocrine chromaffin cells.

Next, calcium levels were measured in individual cells in adrenal slices from Lyz2cre-GCaMP6f-tdTomato mice. Under basal conditions, calcium levels were stable and spontaneous transients were rarely seen. I initially examined the response to purinergic agonists because ATP is a transmitter that is released from chromaffin cells (Hollins and Ikeda, 1997; Zhang et al., 2019), and P2Y receptors are widely expressed in macrophages (Lovászi et al., 2021). ATP had only modest effects on [Ca^2+^]_i_, but application of UDP (an agonist of P2Y6 and P2Y14 receptors) consistently increased calcium levels in adrenal macrophages (Fig. 6*B*). Only a few cells responded to UDP-glucose (P2Y14 agonist) while the actions of UTP were comparable with UDP. Other purinergic agonists, adenosine and ADP, were ineffective (Fig. 6*B*). The response to 100 µM UDP was inhibited by the P2Y receptor antagonist reactive blue-2 (Fig. 6*C*). This pharmacological profile is consistent with expression of the P2Y6 receptor (Jacobson et al., 2020) in adrenal medulla macrophages, in agreement with RNA-seq studies (Bedoya-Reina et al., 2021; Dolfi et al., 2022).

**Figure 7.**
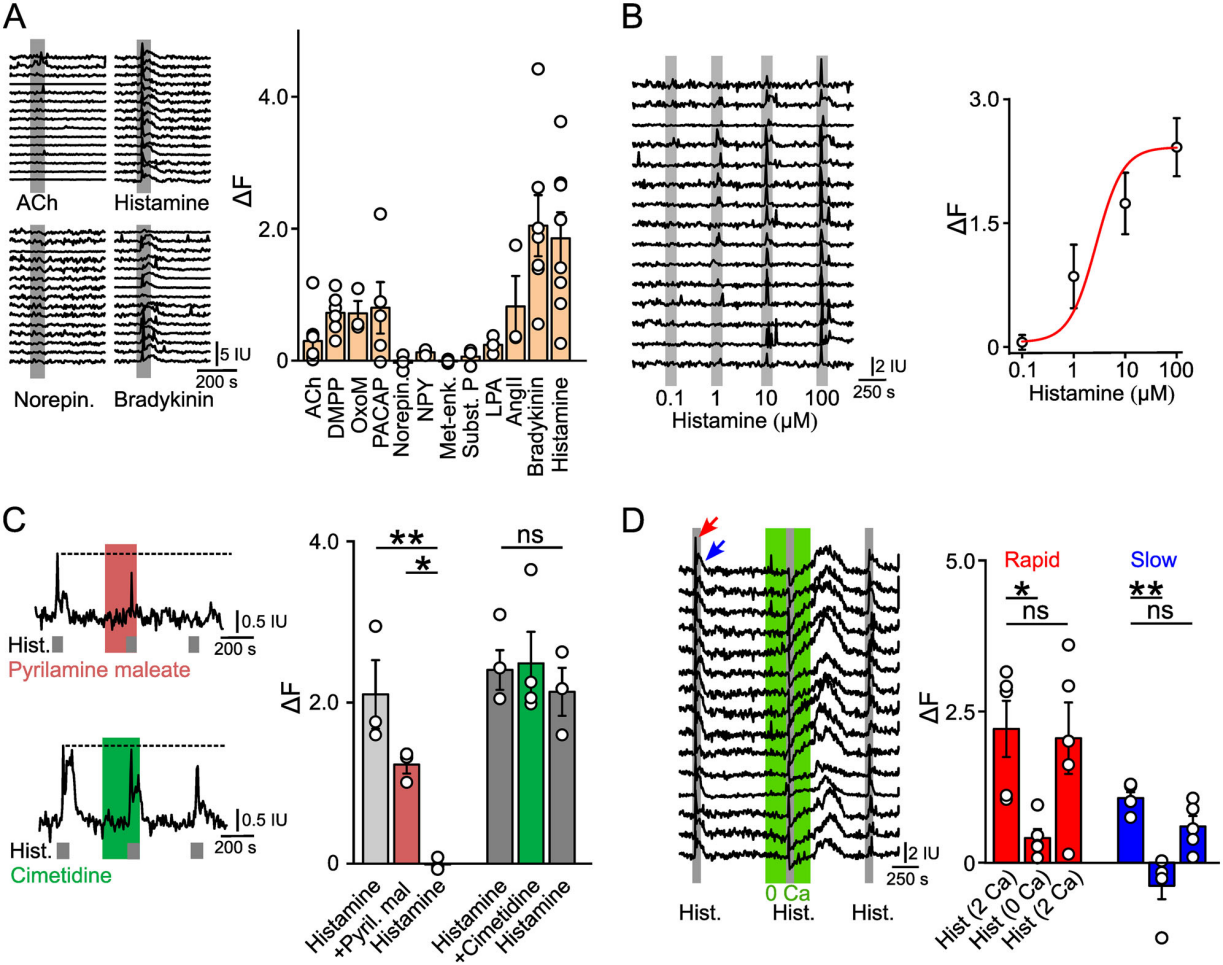
Multiple GPCR agonists increase calcium levels in adrenal macrophages. ***A***, Examples of GCaMP6f fluorescence changes in medulla macrophages in response to 100 µM ACh, 100 µM histamine, 100 µM norepinephrine, and 1 µM bradykinin (left panel). Group data quantifying the change in GCaMP6f signal in response to multiple agonists (right panel; mean ± SEM, *n* = 3–9 mice, 15 cells per mouse, 100 µM ACh, norepinephrine, oxotremorine-M, histamine; 50 µM DMPP; 10 µM LPA; 1 µM NPY, substance P, angiotensin II, bradykinin, PACAP, met-enkephalin). ***B***, GCaMP6f fluorescence signal in macrophages in an adrenal slice in response to histamine application (left panel). Group data (right panel; mean ± SEM, *n* = 3–5 mice, 15 cells per mouse; EC_50_ ∼2.7 µM). ***C***, Calcium increase in medulla macrophages in response to 10 µM histamine is blocked by 0.1 µM pyrilamine maleate (H1 receptor antagonist), but not by 10 µM cimetidine (H2 receptor antagonist). Left panel, each trace is the average of 15 cells in a single adrenal slice). Group data quantifying the effect (right panel; mean ± SEM, *n* = 3–4 mice, 15 cells per mouse). ***D***, Sample records showing the response to 100 µM histamine is reduced in the absence of external calcium (left panel, 15 cells in a single adrenal slice, red and blue arrows indicate the rapid and slow phases of the histamine-evoked increase in GCaMP6f signal). Group data quantifying the histamine-evoked increase in GCaMP6f signal (right panel; mean ± SEM, *n* = 5 mice, 15 cells per mouse). *p < 0.05, **p < 0.01. See also Extended Data Figure 7-1 for more details.

Acetylcholine is the classical transmitter released from preganglionic neurons that innervate chromaffin cells (Barbara and Takeda, 1996). Application of 100 µM and 1 mM ACh both generated a biphasic increase in [Ca^2+^]_i_ in adrenal macrophages (Fig. 7*A*; Extended Data Fig. 7-1*A,B*). Agonists of nACh receptors including dimethylphenylpiperazinium (DMPP), which is widely used to activate adrenal nAChRs (Brindley et al., 2017; Chen et al., 2024; Maldifassi et al., 2024), were particularly effective (Fig. 7*A*; Extended Data Fig. 7-1*C,D*). Oxotremorine-M (a muscarinic agonist) also increased [Ca^2+^]_i_ (Fig. 7*A*). The effect of ACh and Oxotremorine-M decremented with repeated application, but the response to DMPP was blocked by the nicotinic antagonist mecamylamine (Extended Data Fig. 7-1*C,D*) confirming the effect was mediated by nACh receptors. PACAP, a preganglionic peptide cotransmitter (Kumar et al., 2010) also increased [Ca^2+^]_i_ in adrenal macrophages (Fig. 7*A*). In contrast, modulators released by chromaffin cells (norepinephrine, NPY, and met-enkephalin), sensory neurons (substance P), and multiple cell types (LPA and glutamate) were without effect (Fig. 7*A*, Extended Data Fig. 7-1*D*). GABA which is synthesized and released from chromaffin cells (Inoue et al., 2010) had a small but consistent effect (Extended Data Fig. 7-1*D*). The vasoactive hormones, angiotensin II and bradykinin, both increased [Ca^2+^]_i_ in medulla macrophages (Fig. 7*A*).

10.1523/ENEURO.0153-24.2025.f7-1 Figure 7-1 **Agonists increase calcium levels in adrenal macrophages.** A. Examples of GCaMP6f fluorescence changes in medulla macrophages in response to varying concentrations of ACh. B. Group data quantifying the change in GCaMP6f signal in response to ACh (mean ± SEM, n = 3 - 9 mice, 15 cells per mouse, 100  µM data is also shown in Figure 7A). C. The biphasic increase in GCaMP6f fluorescence evoked by the nicotinic agonist DMPP (50  µM) is blocked by the antagonist mecamylamine (100  µM). Application of histamine (100  µM) shows that the cells remain responsive. D. Group data showing the change in GCaMP6f fluorescence in response to 5  µM and 50  µM DMPP; 50  µM DMPP ± 100  µM mecamylamine; 100 and 1000  µM GABA and glutamate (mean ± SEM, n = 3 - 6 mice, 15 cells per mouse). * P < 0.05, *** P < 0.001. Download Figure 7-1, TIF file.

Finally, application of histamine also reliably increased [Ca^2+^]_i_ (Fig. 7*A*) in medulla macrophages with a threshold dose of ∼100 nM (Fig. 7*B*). The effect of histamine was mediated by H1 receptors because it was blocked by pyrilamine maleate but not by cimetidine, an H2 receptor antagonist (Fig. 7*C*). The histamine-induced increase in [Ca^2+^]_i_ was biphasic with a rapid initial peak and slower second phase (Fig. 7*C*,*D*). Removal of extracellular calcium reduced the amplitude of both phases (Fig. 7*D*) consistent with the involvement of both calcium influx and release from internal stores. When calcium was returned to the bathing medium, there was a large rebound increase in [Ca^2+^]_i_ which is characteristic of store-operated calcium entry (Fig. 7*D*).

**Figure 8.**
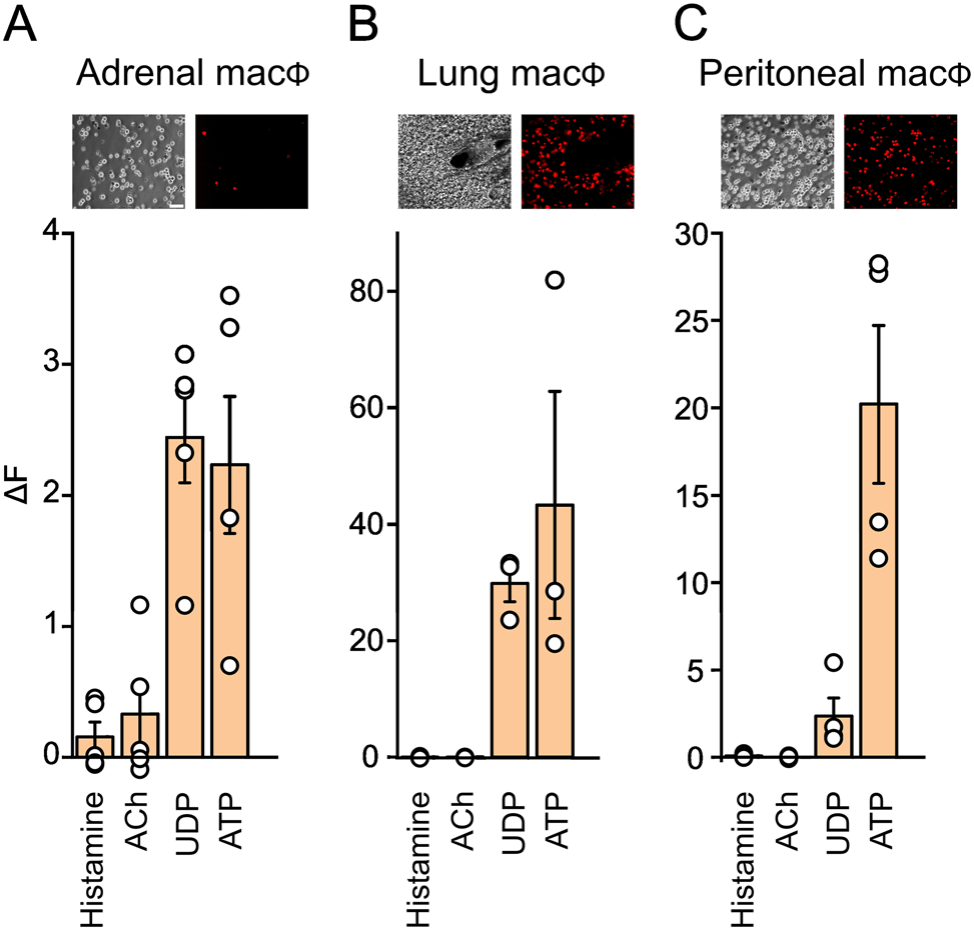
Isolation of adrenal macrophages alters the calcium increase in response to GPCR agonists. ***A***, Quantification of the change in GCaMP6f signal in adrenal macrophages in vitro, in response to 100 µM histamine, ACh, UDP, and ATP. In contrast to the response in slices, isolated macrophages do not respond to histamine and ACh. ***B***, Lung macrophages in slices and ***C***, peritoneal macrophages in vitro respond to UDP and ATP but not to histamine or ACh. Top images in ***A−C*** show corresponding phase contrast images and RFP fluorescence, respectively. Mean ± SEM, *n* = 3–5 mice, 15 cells per mouse. Scale bar 50 µm.

The robust response of adrenal macrophages to ACh and multiple GPCR (G protein-coupled receptor) agonists was unexpected. However, because the adrenal is composed of numerous cell types, it is possible that these effects are mediated indirectly. I examined this issue in two ways. First, I minimized the contribution of other cells by dissociating adrenal tissue from Lyz2cre-GCaMP6f mice and examined the response of tdTomato-expressing cells in vitro. Four agonists were tested that either evoked responses ex vivo (UTP, histamine, and ACh) or were expected to do so (ATP). Application of UDP and ATP increased [Ca^2+^]_i_, but surprisingly, histamine and acetylcholine were now ineffective (Fig. 8*A*; histamine *p* = 0.239; ACh *p* = 0.233; one-sample *t* test). Curiously, ATP reliably increased [Ca^2+^]_i_ in vitro in contrast to the results in slices (compare Figs. 6*B*, 8*A*), perhaps because the cultured cells are a mix of cortical and medulla macrophages (autofluorescence prevented quantification of [Ca^2+^]_i_ in cortical macrophages in slices). Second, I tested whether other peripheral macrophages showed similar responses to the same agonists. Both peritoneal and pulmonary macrophages from Lyz2cre-GCaMP6f mice responded to UDP and ATP but not to ACh or histamine (Fig. 8*B*,*C*). These results confirm that UDP (and ATP) act directly on adrenal macrophages, but the effect of histamine (and perhaps ACh) are likely mediated via an intervening cell type(s).

## Discussion

While it is accepted that the adrenal gland contains multiple types of immune cells, their zonal location and function is less clear. This issue is significant because the adrenal is broadly composed of two regions, an outer cortex containing endocrine cells and an inner medulla that is neuroendocrine and part of the sympathetic nervous system. Given the function of these regions is specialized, it seems likely that the functions of the resident immune cells will also be distinct. As an initial step toward understanding their role in adrenal physiology, I investigated immune cell distribution in the mouse adrenal medulla and the response to a range of adrenal-relevant neuromodulators (Carbone et al., 2019). This region of the gland was found to contain a large population of CD45-immunoreactive myeloid cells, most of which appear to be F4/80+/Lyz2+ macrophages. These cells are found throughout the gland, but within the medulla, they reside close to multiple cell types including chromaffin cells, endothelial cells, satellite glial cells, and presumptive preganglionic axons. Thus, like the CNS which contains microglia, the neuroendocrine component of the adrenal gland is also populated with resident macrophages. This finding confirms earlier studies in both rodents and humans which showed that macrophages are present within all four adrenal zones (Hume et al., 1984; González-Hernández et al., 1994; Sato, 1998; Schober et al., 1998; Engström et al., 2008). A recent RNA-seq study of mouse adrenal gland immune cells identified four macrophage populations but whether there is a medulla-specific population is not known (Dolfi et al., 2022).

Monitoring the levels of intracellular calcium in medulla macrophages using Lyz2cre-GCaMP6f mice showed these cells can respond to a wide variety of modulators that are generated within the gland or found within the systemic circulation. In common with many macrophage populations (Hanley et al., 2004; del Rey et al., 2006; Koizumi et al., 2007; Weitz et al., 2018; Gryshchenko et al., 2021), those within the medulla are sensitive to multiple purinergic agonists. In slices, the largest effects were to uridine nucleotides. Combined with the modest response to adenosine nucleotides, the receptor most likely to be responsible is P2Y6, and this is also consistent with inhibition of the UDP-evoked response by reactive blue-2 (although it is acknowledged that some agonists were only tested at a single concentration). P2Y6 receptors are expressed by a diversity of macrophages including microglia and peritoneal macrophages (del Rey et al., 2006; Koizumi et al., 2007; Bar et al., 2008; Morioka et al., 2013). In microglia, activation of P2Y6 receptors by UDP released from damaged neurons increases microglial phagocytosis (Koizumi et al., 2007). Although this pathway could also be active in the adrenal, chromaffin cells release ATP as a cotransmitter (Hollins and Ikeda, 1997; Zhang et al., 2019), and UTP and UDP are found in dense core secretory granules in chromaffin cells (Van Dyke et al., 1977; Bankston and Guidotti, 1996). Thus, medulla macrophages are likely to be exposed to high concentrations of secreted purines under physiological levels of sympathetic activity. Unexpectedly ATP had little effect on [Ca]_i_ on adrenal macrophages in slices but evoked a substantial response in vitro. The reason for this is not known. One possibility is that in the slice, macrophage receptors are partially desensitized due to tonic ATP release from chromaffin cells. Another possibility is that ATP primarily acts as a paracrine factor on chromaffin cells rather than on macrophages. Finally, CNS macrophages (microglia) show rapid transcriptional changes when placed in vitro (Bohlen et al., 2017), and thus there may not be a complete correspondence between the responses of macrophages in vitro and in the adrenal slice.

A number of other agonists also effectively increased calcium in medulla macrophages including ACh and PACAP which are classical and peptidergic cotransmitters, respectively, released by the preganglionic neurons that innervate chromaffin cells (Barbara and Takeda, 1996; Inoue et al., 2000; Kumar et al., 2010). Curiously the response to ACh was absent when adrenal macrophages were isolated in vitro. This suggests that the receptors are either rapidly lost when the cells were placed in culture or that the actions of ACh are mediated via an intervening cell type. A similar discrepancy was noted for histamine which robustly increased calcium in medulla macrophages in adrenal slices but had no significant effect on adrenal macrophages in vitro. The identity of a putative intervening cell type(s) is unknown, although chromaffin cells are plausible candidates since they express nicotinic and muscarinic ACh receptors (Akaike et al., 1990; Barbara and Takeda, 1996; Barbara et al., 1998; Colomer et al., 2010). Suggestively, chromaffin cells also express H1 histamine receptors, the same subtype involved in the macrophage response to histamine (Marley et al., 1991; Borges, 1994; Currie and Fox, 2000). Endogenous sources of ACh include the sympathetic preganglionic neurons (Barbara and Takeda, 1996). Histamine is not known to be secreted from preganglionic neurons but has been reported in chromaffin cells (Häppölä et al., 1985; Tuominen et al., 1993). Mast cells are present in the adrenal, but these are in the cortex (Kim et al., 1997; Boyer et al., 2017) and so unlikely to supply histamine to macrophages in the medulla. Staining for c-kit, a marker of mast cells (Tauber et al., 2023), confirmed these cells are absent from the mouse adrenal medulla (data not shown). Previous work has also suggested that a likely origin of histamine within the adrenal is entry from the systemic circulation (Borges, 1994). The threshold dose for the histamine-induced increase in cytoplasmic calcium in medulla macrophages was ∼100 nM. Systemic levels of histamine are in the low nanomolar range although they can rise several fold during an inflammatory response (Shiva et al., 2008; Boehm et al., 2019).

Although the role of the medulla macrophages remains to be determined, their close association with the preganglionic input and the postganglionic chromaffin cells raises the possibility they modulate sympatho-adrenal activity. Conceivably this could occur during a classical immune response (such as infection), under fight-or-flight conditions (such as chronic stress), or on a developmental time scale (such as synaptic pruning). Macrophages are known to be involved in all of these contexts (Wolf et al., 2017; Pavlov et al., 2018; Meroni et al., 2019; Biltz et al., 2022). The mechanism(s) underlying this communication is not, clear but adrenal macrophages can synthesize a variety of cytokines, including IL-1β, TNFα, TGF-β, and other inflammatory molecules (González-Hernández et al., 1996; Engström et al., 2008; Xu et al., 2024). Many of these signaling molecules regulate the synthesis and release of catecholamines and neuropeptides from chromaffin cells (Rosmaninho-Salgado et al., 2009; Douglas et al., 2010; Jewell et al., 2011; Carbone et al., 2019).

Finally, although macrophages are distributed throughout the adrenal medulla, they were often associated with axonal processes (Fig. 4*B*). This is reminiscent of the NAMs that are found in the lungs (Ural et al., 2020), adipose tissue (Pirzgalska et al., 2017), skin (Kolter et al., 2019), and GI tract (Muller et al., 2014). In future work it will be informative to test whether adrenal macrophages are able to acutely modulate epinephrine release and sympatho-adrenal function.

## Synthesis

Reviewing Editor: Viji Santhakumar, University of California Riverside Decisions are customarily a result of the Reviewing Editor and the peer reviewers coming together and discussing their recommendations until a consensus is reached. When revisions are invited, a fact-based synthesis statement explaining their decision and outlining what is needed to prepare a revision will be listed below. The following reviewer(s) agreed to reveal their identity: Ricardo Borges, Arturo Hernandez-Cruz.

The authors have appropriately addressed all reviewer concerns
