# Supplementary figures and images for: Mouse Adrenal Macrophages Are Associated with Pre- and Postsynaptic Neuronal Elements and Respond to Multiple Neuromodulators
Source: eNeuro. 2025 Feb 18;12(2):ENEURO.0153-24.2025. doi: 10.1523/ENEURO.0153-24.2025 (PMC11856350; doi:10.1523/ENEURO.0153-24.2025)

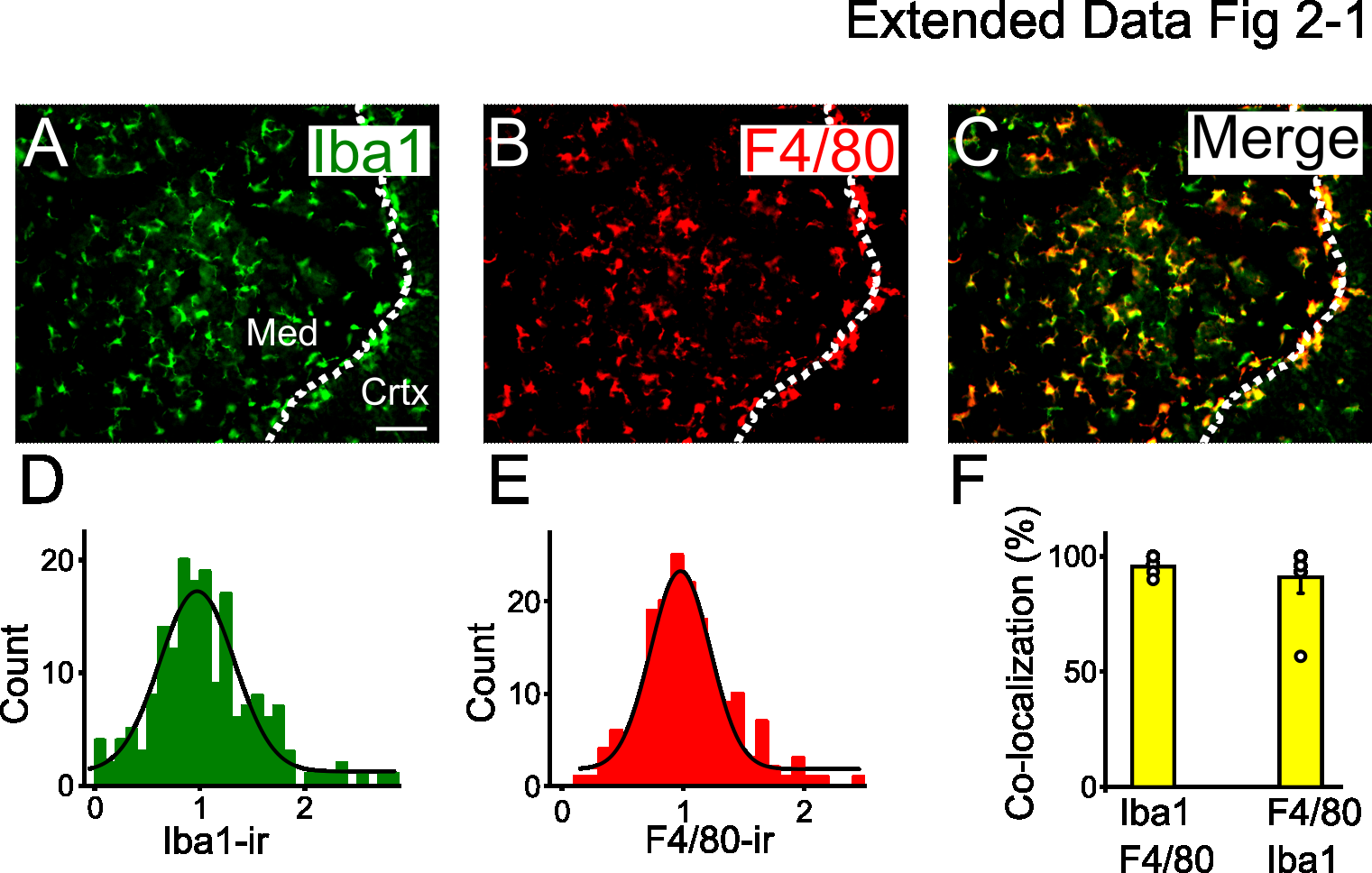

Supplement: Figure 2-1 — Macrophages in the adrenal medulla. A-C. Adrenal cryosections from a wild type mouse co-stained for Iba1 and F4/80. D. Distribution of fluorescence intensities for Iba1-ir cells fit with a single Gaussian distribution. E. Distribution of F4/80-ir fluorescent signal. F. Group data showing that Iba1- and F4/80-ir cells in the medulla are co-localized (mean ± SEM, n = 6 mice). Scale bar 50 µm. Download Figure 2-1, TIF file. [file eneuro-12-ENEURO.0153-24.2025-s002.tif]

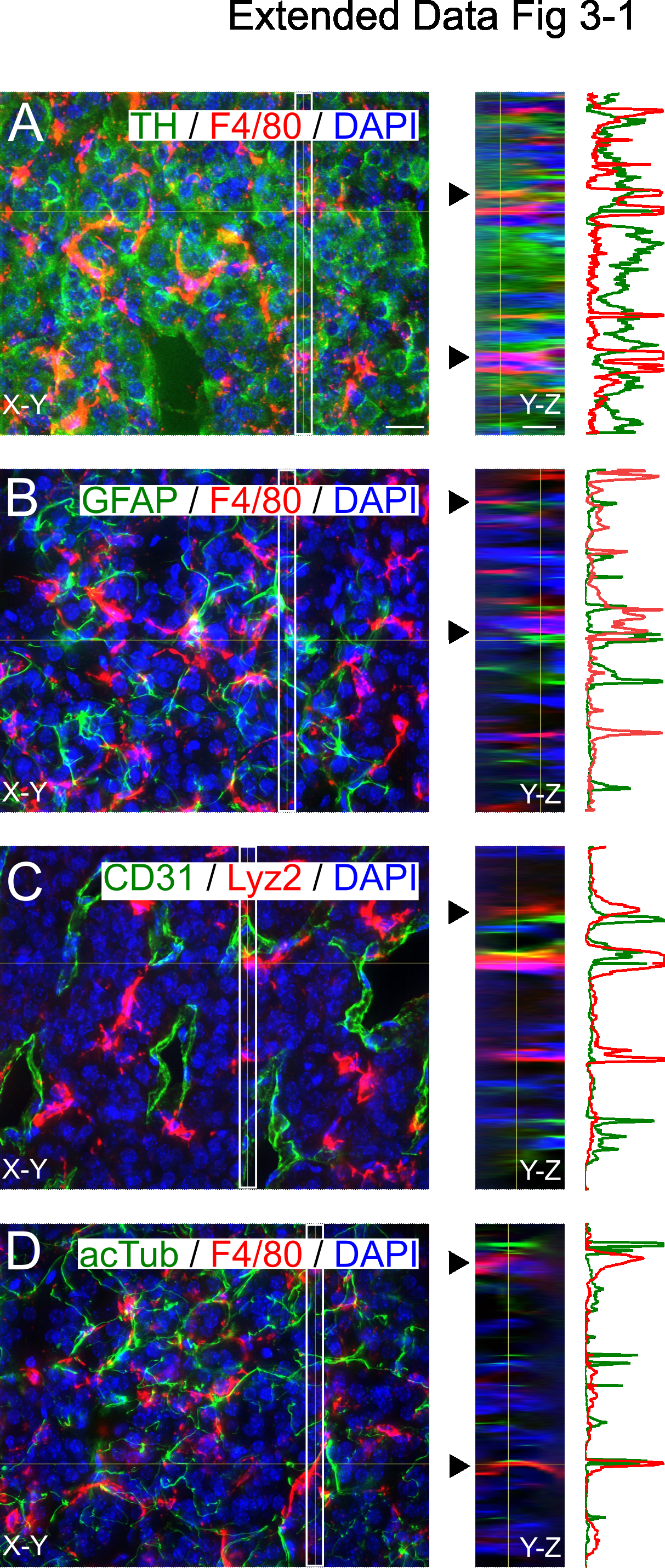

Supplement: Figure 3-1 — Macrophages in the adrenal medulla are close to multiple adrenal cell types. A. F4/80-ir macrophages intermingle with TH-ir chromaffin cells (left panel). In the orthogonal view (middle panel), areas of close juxtaposition (arrow heads) are seen. Line scans of fluorescence intensity normalized to peak intensity in each channel (right panel). B. F4/80-ir macrophages and GFAP-ir satellite glial cells. C. Some RFP-ir macrophages in a Lys2-cre-GCaMP6f-tdTomato mouse are close to CD31-ir endothelial cells. D. Some F4/80-ir macrophages are aligned along acTub-ir neuronal processes. In A-D, orthogonal views are taken from the white boxed areas shown in the corresponding left panels. Line scans were measured along the yellow vertical line shown in each Y-Z projection. Scale bar 20 µm (left panels); 5 µm (z axis, middle panels). Download Figure 3-1, TIF file. [file eneuro-12-ENEURO.0153-24.2025-s003.tif]

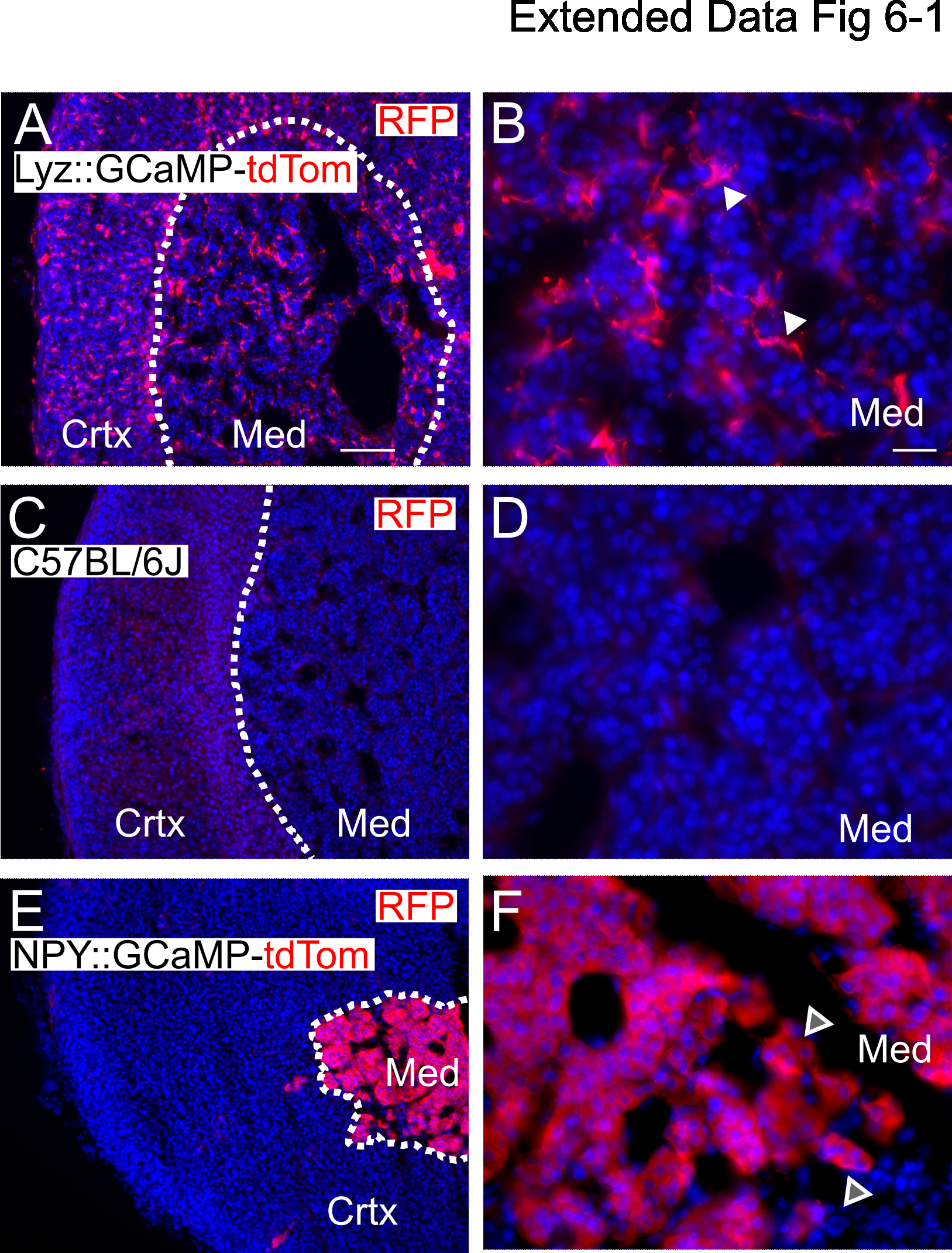

Supplement: Figure 6-1 — Controls for specific labelling of immune cells in the mouse adrenal gland. A. RFP-immunoreactive cells in the adrenal gland of a Lys2-cre-GCaMP6f-tdTomato mouse. B. Higher power image showing RFP-ir cells (arrow heads) in the adrenal medulla. C. RFP-ir in the adrenal gland of a wild type mouse. D. Higher power view of the adrenal medulla showing that background levels of fluorescence are low. Thus, fluorescence signal in A,B does not arise from non-specific secondary antibody staining. E. RFP-ir in the adrenal gland of a NPYcre-GCaMP6f-tdTomato mouse. F. RFP-ir cells in the adrenal medulla with the characteristic morphology of chromaffin cells (arrow heads). No cells with a macrophage-like morphology (compare with B) are seen. Scale bar 200 µm (A,C,E); 20 µm (B,D,F). Download Figure 6-1, TIF file. [file eneuro-12-ENEURO.0153-24.2025-s004.tif]

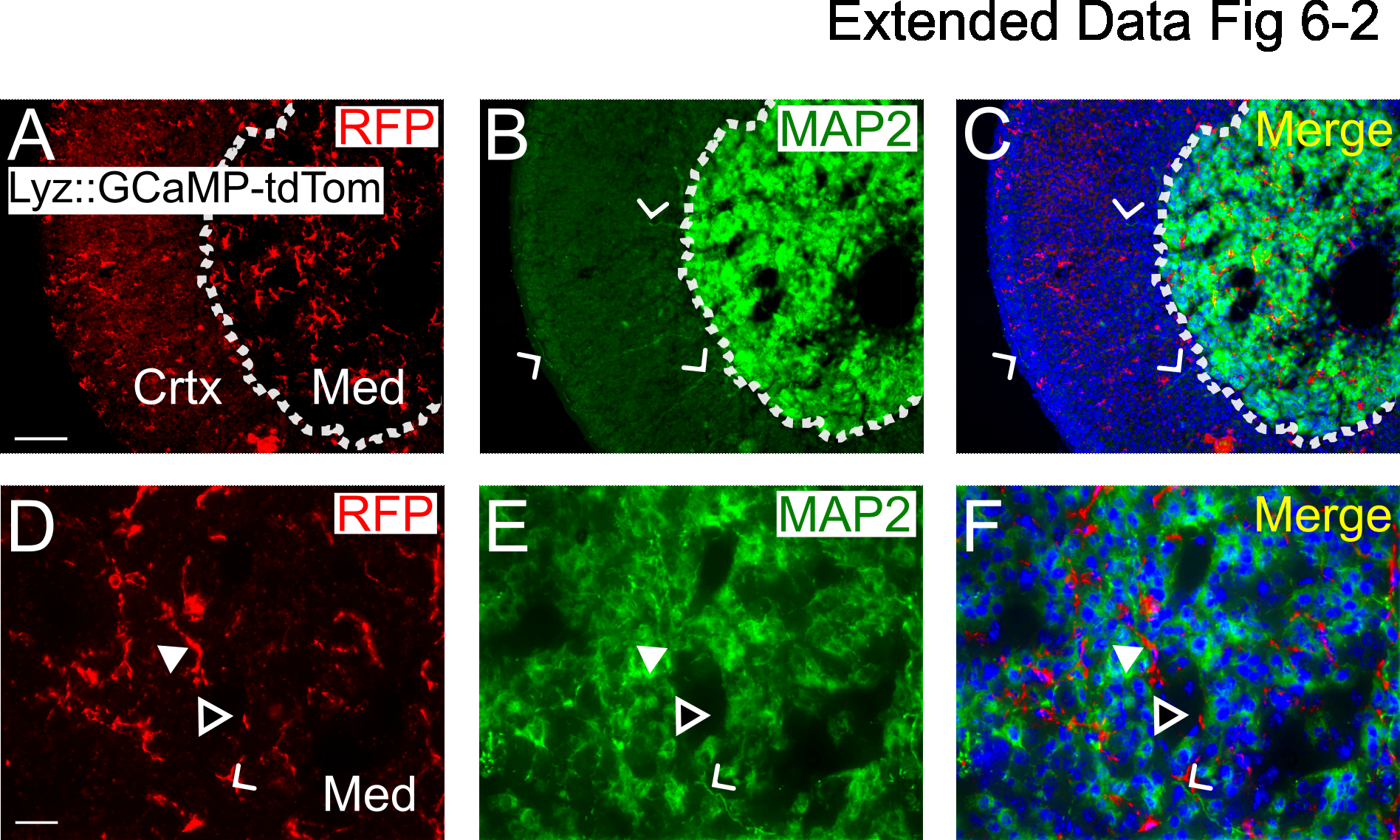

Supplement: Figure 6-2 — No neuronal expression of GCaMP6f is seen in the adrenal medulla of Lyz2cre-GCaMP6f-tdTomato mice. A. RFP-ir in the adrenal medulla of a Lyz2cre-GCaMP6f-tdTomato mouse. B. MAP2-ir (a neuronal marker) in the same cryosection is present in cells throughout the adrenal medulla (presumably chromaffin cells and intra-adrenal ganglion neurons) and in cell processes in the capsule and cortex (arrow heads). C. Merged image. D-F. In the adrenal medulla, higher power images show that RFP-ir cells (white arrowhead) do not co-localize with MAP2-ir cells (filled arrowhead) or MAP2-ir processes (open arrowhead). Combined with other data (Fig 2–4, 6) this indicates that RFP (and thus GCaMP6f) is expressed in macrophages and not neuronal cells. Scale bar 200 µm (A,B,C); 20 µm (D,E,F). Download Figure 6-2, TIF file. [file eneuro-12-ENEURO.0153-24.2025-s005.tif]

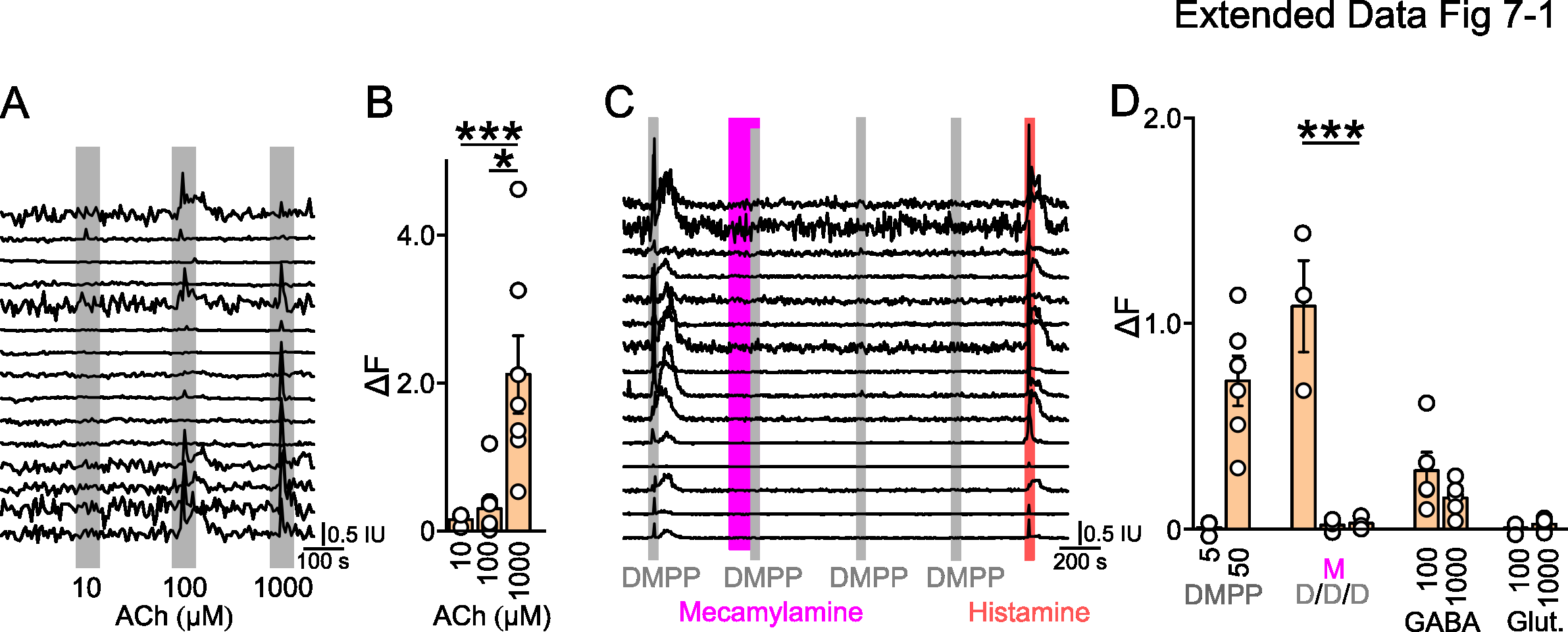

Supplement: Figure 7-1 — Agonists increase calcium levels in adrenal macrophages. A. Examples of GCaMP6f fluorescence changes in medulla macrophages in response to varying concentrations of ACh. B. Group data quantifying the change in GCaMP6f signal in response to ACh (mean ± SEM, n = 3 - 9 mice, 15 cells per mouse, 100 µM data is also shown in Figure 7A). C. The biphasic increase in GCaMP6f fluorescence evoked by the nicotinic agonist DMPP (50 µM) is blocked by the antagonist mecamylamine (100 µM). Application of histamine (100 µM) shows that the cells remain responsive. D. Group data showing the change in GCaMP6f fluorescence in response to 5 µM and 50 µM DMPP; 50 µM DMPP ± 100 µM mecamylamine; 100 and 1000 µM GABA and glutamate (mean ± SEM, n = 3 - 6 mice, 15 cells per mouse). * P < 0.05, *** P < 0.001. Download Figure 7-1, TIF file. [file eneuro-12-ENEURO.0153-24.2025-s006.tif]
